# The deletion of the ORF1 and ORF71 genes reduces virulence of the neuropathogenic EHV-1 strain Ab4 without compromising host immunity in horses

**DOI:** 10.1371/journal.pone.0206679

**Published:** 2018-11-15

**Authors:** Christine L. Wimer, Christiane L. Schnabel, Gillian Perkins, Susanna Babasyan, Heather Freer, Alison E. Stout, Alicia Rollins, Nikolaus Osterrieder, Laura B. Goodman, Amy Glaser, Bettina Wagner

**Affiliations:** 1 Department of Population Medicine and Diagnostic Sciences, College of Veterinary Medicine, Cornell University, Ithaca, New York, United States of America; 2 Department of Clinical Sciences, College of Veterinary Medicine, Cornell University, Ithaca, New York, United States of America; 3 Institut für Virologie, Freie Universität Berlin, Berlin, Germany; Instituto Butantan, BRAZIL

## Abstract

The equine herpesvirus type 1 (EHV-1) ORF1 and ORF71 genes have immune modulatory effects *in vitro*. Experimental infection of horses using virus mutants with multiple deletions including ORF1 and ORF71 showed promise as vaccine candidates against EHV-1. Here, the combined effects of ORF1 and ORF71 deletions from the neuropathogenic EHV-1 strain Ab4 on clinical disease and host immune response were further explored. Three groups of EHV-1 naïve horses were experimentally infected with the ORF1/71 gene deletion mutant (Ab4ΔORF1/71), the parent Ab4 strain, or remained uninfected. In comparison to Ab4, horses infected with Ab4ΔORF1/71 did not show the initial high fever peak characteristic of EHV-1 infection. Ab4ΔORF1/71 infection had reduced nasal shedding (1/5 vs. 5/5) and, simultaneously, decreased intranasal interferon (IFN)-α, interleukin (IL)-10 and soluble CD14 secretion. However, Ab4 and Ab4ΔORF1/71 infection resulted in comparable viremia, suggesting these genes do not regulate the infection of the mononuclear cells and subsequent viremia. Intranasal and serum anti-EHV-1 antibodies to Ab4ΔORF1/71 developed slightly slower than those to Ab4. However, beyond day 12 post infection (d12pi) serum antibodies in both virus-infected groups were similar and remained increased until the end of the study (d114pi). EHV-1 immunoglobulin (Ig) G isotype responses were dominated by short-lasting IgG1 and long-lasting IgG4/7 antibodies. The IgG4/7 response closely resembled the total EHV-1 specific antibody response. *Ex vivo* re-stimulation of PBMC with Ab4 resulted in IFN-γ and IL-10 secretion by cells from both infected groups within two weeks pi. Flow cytometric analysis showed that IFN-γ producing EHV-1-specific T-cells were mainly CD8^+^/IFN-γ^+^ and detectable from d32pi on. Peripheral blood IFN-γ^+^ T-cell percentages were similar in both infected groups, albeit at low frequency (~0.1%). In summary, the Ab4ΔORF1/71 gene deletion mutant is less virulent but induced antibody responses and cellular immunity similar to the parent Ab4 strain.

## Introduction

Equine herpesvirus type-1 (EHV-1) is highly prevalent in the equine population with most horses becoming infected as juveniles and remaining latently infected for life [1]. Latently infected horses act as a virus reservoir. EHV-1 spreads through respiratory secretions and nose-to-nose contact or via fomites. EHV-1 first infects the respiratory epithelium, causing fever and rhinopneumonitis. The virus quickly enters local lymphoid tissues, is spread systemically via a cell-associated viremia, and latency is established in neurons of the trigeminal ganglia [2,3]. Disease manifestations range from subclinical to severe respiratory infection, abortion, neonatal foal death, or equine herpesvirus myeloencephalopathy (EHM) [1,4]. Arteriolar vasculitis and subsequent thrombosis and ischemia causes both the abortigenic and neurologic manifestations [1, 5, 6]. The virus can be reactivated and shed during stress, and may lead to any of the clinical manifestations [1, 7]. Moreover, previously exposed, susceptible horses respond to experimental infection with EHV-1 similar to EHV-1 naïve horses [8]. Through lost time for training and competing, treatment, quarantine, abortion, and death, EHV-1 has great medical and economic impact [1, 9]. In the past 20 years, the increased incidence of morbidity and mortality due to the neurologic manifestation has prompted heightened biosecurity and resurgence in EHV-1 vaccine research [4, 10, 11].

A combination of humoral and cell mediated immunity is believed to be necessary to protect horses from severe clinical disease and to reduce viral shedding [12, 13]. Limiting viremia is assumed to prevent severe disease outcomes, as viremia is associated with the spread of the virus to vascular endothelial cells resulting in abortions or EHM [2, 6, 14, 15, 16]. Cell mediated immunity is believed to be critical for clearance of virus-infected cells [2, 15, 16]. The latter is supported by the finding that increased numbers of EHV-1 specific cytotoxic T cell (CTL) precursors correlated with protection from development of EHM upon experimental challenge infection in older mares [17]. In addition, EHV-1 specific interferon (IFN)-γ producing T helper 1 (Th1) cells were increased in horses that survived a neurological outbreak [18]. Recovering from natural or experimental infection is associated with a broad IgG response dominated by IgG4/7 antibody isotypes [18, 19]. However, high antibody responses of fast onset in combination with low and slowly increasing T-cell immunity are characteristic for young horses overcoming EHV-1 infection and respiratory disease [8]. This supports that protection from all clinical presentations of EHV-1 is likely complex and requires intact humoral and cellular immunity to contain local infection, preclude viral shedding and systemic cell-associated viremia, and prevent clinical disease including abortions and EHM.

Currently, several EHV vaccines are available including two single component inactivated vaccines for prevention of abortion in pregnant mares, a modified-live vaccine (MLV) and different multi-component inactivated vaccines for prevention of respiratory disease [16, 18, 19, 20, 21]. These vaccines and improved biosecurity have reduced the incidence of abortion storms, but neurologic disease outbreaks are still occurring [16, 18, 20, 21]. Two controlled vaccination studies compared the MLV (Rhinomune, Boehringer Ingelheim) with one of the available inactivated vaccines (Flu-vac Innovator 6 combination vaccine, Fort Dodge or Pneumabort-K, Pfizer Animal Health) [16, 20]. All vaccines afforded some protection from clinical signs, nasal shedding and viremia [16, 20]. The inactivated combination vaccine induced stronger initial antibody responses with increased IgG3/5 antibodies, while the MLV predominately resulted in IgG4/7 production [16]. In this controlled study, horses with high IgG3/5:IgG4/7 ratios had increased severity of neurological signs, whereas horses with high IgG4/7 antibodies and low IgG3/5:IgG4/7 ratios did not develop neurological signs or viral shedding [16, 20, 22]. A more recent EHV vaccine study used one of the inactivated vaccines (Calvenza EHV, Boehringer Ingelheim). A novel quantitative EHV-1 multiplex approach to determine anti-EHV-1 antibodies showed that the humoral response to this vaccination was dominated by high initial IgG1 and long-lasting IgG4/7 isotypes, while only minor amounts of IgG3/5 and other isotypes were induced [21]. A similar antibody isotype pattern was identified after experimental infection of weanlings, which resulted in mild respiratory disease [8]. In summary, the previous studies suggest that an EHV-1 specific IgG response dominated by IgG4/7 is associated with protection and effective virus neutralization [16, 18].

Although overall more protective than the inactivated vaccine, some horses vaccinated with the MLV Rhinomune in the trial of Goodman et al. [16] still shed virus and several became viremic when challenge infected, indicating that vaccination with available MLV is not fully sufficient for sterile immunity [18]. In another study on correlates of immunity to EHV-1, previously infected horses that survived an outbreak and were vaccinated with MLV had higher SN-titers and stronger cellular immunity to EHV-1 than MLV vaccinated horses that had not been infected. This comparison suggests, that immunity induced by currently applied vaccination may be weaker than that induced by infection. It is accordingly possible that the response pattern to available vaccines and EHV-1 infection is similar in general, but the strength and durability may vary. Accordingly, EHV-1 infection appears the most immunogenic scenario to date.

As protection by available vaccines seems not sufficient to prevent EHM outbreaks in the US, several experimental MLV vaccine strains have been examined aiming to identify safe candidates with improved immunogenicity. The identification of immune modulatory virulence factors of EHV-1 seems critical to achieve this. A laboratory strain of EHV-1 (KyA), containing six gene deletions, was found to be avirulent in young horses. While antibody responses to intranasal inoculation was limited, it reduced clinical signs, nasal shedding and viremia following challenge infection [23]. It was postulated that clinical protection in the face of a low antibody response was due to cellular immune responses. This led to the examination of a modified-live virus missing only two of the six genes deleted in KyA, the gE and gI genes. Intranasal vaccination of horses with the resulting virus provided partial protection from clinical signs but failed to reduce viral shedding or viremia. This vaccine candidate also induced only low serum neutralizing (SN) antibody responses indicating poor immunogenicity of the gE/gI deletion mutant virus [24]. More recently, two other genes missing in the KyA strain were examined, ORF1 and ORF2 [25]. One, of them, the ORF1 gene was explored *in vitro* and shown to encode a trans membrane protein–pUL56– that is localized to the Golgi apparatus of host cells [26]. ORF1 contributed to immune evasion *in vitro* by down-regulation of major histocompatibility complex class I (MHC-I), modulation of IFN-α and IL-10 expression, and down-regulation of chemokine expression limiting chemotaxis of monocytes and neutrophils [26, 27]. A double deletion mutant lacking the ORF1 and ORF2 genes of the neuropathogenic EHV-1 strain Ab4 was designed and used in an *in vivo* infection study in ponies. The ORF1/2 deletion reduced the initial pyrexia and resulted in shorter duration and lower viral nasal shedding compared to the parent Ab4 strain. The double deletion also provoked increased IgG1 and IgG3/5 production and enhanced mRNA expression of Tbet and IL-8 in peripheral blood mononuclear cells (PBMC). The results suggested an altered ability of the Ab4ΔORF1/2 deletion mutant virus to modulate the immune response and pointed to one or both of these gene products as contributors to immune evasion, namely as immune modulatory virulence factors of EHV-1 [25].

The avirulent strain KyA also harbors an in frame partial gene deletion in gene 71 (ORF71), the gene encoding glycoprotein 2 [28, 29, 30, 31, 32]. Envelope glycoproteins play vital roles in herpesvirus infection and propagation, and are important targets of the host immune system. EHV-1 has 13 different glycoprotein genes and 11 of these are conserved in the *Alphaherpesvirinae* subfamily [33, 34]. However, homologs of EHV-1 ORF71 are only known to exist in EHV-4, EHV-9 and Asinine herpes virus [35, 36]. ORF71 plays a fundamental role in EHV-1 pathogenesis in a mouse model and in virus propagation as demonstrated *in vitro* [37, 38]. In the absence of ORF71 EHV-1 binds to cells less effectively *in vitro*, is impaired in maturation and envelopment, and egresses from the cell less efficiently [37, 39, 40]. Cell-to-cell spread of the virus is unaffected and is the primary means of propagation in the absence of ORF71 [37, 39, 40]. The KyA ORF71 gene encodes a markedly truncated protein that is not functionally equivalent to the full-length counterpart [31]. When the full-length ORF71 is restored in strain KyA normal pathological responses are noted in the lower respiratory tract of mice [38]. When studied as a vaccine candidate, Ab4 with an ORF71 deletion was able to confer protection against pulmonary disease following challenge with the wild type virus in mice [41]. However, the effects of the deletion of ORF71 as a potential virulence factor during EHV-1 infection in the horse has not yet been evaluated.

Until today, none of the available vaccines induces sufficient immunity to prevent EHV-1 associated febrile disease, nasal virus shedding, and viremia completely [16, 18, 20]. An ideal vaccine against EHV-1 should provide strong immunity against the virus that protects from clinical signs, nasal virus shedding and viremia. Comparison of the immune response induced by new vaccine candidates to that induced by wild-type EHV-1 infection, the strongest immunogenic available to date, provides a meaningful evaluation of immunogenicity.

In this approach we have used a double gene deletion mutant of Ab4 (Ab4ΔORF1/71) missing both, ORF1 and ORF 71, for experimental infection of EHV-1 naïve horses in comparison with the parent Ab4 strain. Based on results from previous *in vitro* experiments or mouse models outlined above, both gene deletions were promising candidates for reducing virulence of the virus, limiting immune evasion and therefore potentially increasing immunity in the natural host, the horse. Here, we explored the effects of the ORF1/71 gene deletion from the EHV-1 Ab4 virus *in vivo* by evaluating the effects of these probable virulence factors on the induction of clinical disease, nasal shedding, viremia and humoral and cellular host immunity in horses.

## Materials and methods

### EHV-1 naïve horses

Fifteen horses from the EHV-1-free herd of Icelandic horses at Cornell University were enrolled in this study [8, 21]. All horses were offspring of the same stallion. They were born and kept as a group in Iceland where EHV-1 has never been reported [42]. The horses were imported to the US in April 2013 when they were 22 months of age. To maintain the EHV-1 free status of the horses, import quarantine was performed at an isolated facility at Cornell University under USDA/APHIS supervision. All horses were kept at this facility and had no contact to other horses in the US prior to and for the duration of this study. The facility had restricted access for people to avoid infection with common US pathogens and to maintain the EHV-1-free status of the Icelandic herd. Personnel caring for the herd did not have contact to other horses or applied biosecurity precautions before entering the EHV-1-controlled facility. After release from importation quarantine, the horses were vaccinated against rabies and tetanus and dewormed but were not vaccinated or treated otherwise. Before EHV-1 infection, the horses were kept on pasture and were clinically healthy. Grass hay was fed to all horses ad libitum. The experimental EHV-1 infection and all sample collections for this study were carried out in accordance with the Guide for the Care and Use of Laboratory Animals of the National Institute of Health as well as the Guide for Care and Use of Animals in Agricultural Research and Teaching. The animal protocol was approved by the Institutional Animal Care and Use Committee at Cornell University (protocol #2011–0011). All efforts were made to minimize suffering of the animals for example by short sedation for nervous or excited horses before sampling. All horses survived the experimental study and were kept at the facility at Cornell University as research horses. The isolation precautions for the EHV-1 controlled herd were maintained in the same way as prior to the experimental infection.

### Parent and deletion mutant virus used for infection

The neuropathogenic EHV-1 strain Ab4 [43] was initially isolated from a quadriplegic mare in an outbreak in 1980 [6, 44]. Ab4 was used here as the parent virus for experimental infection. The ORF1/71 gene deletion mutant (Ab4ΔORF1/71) was previously described in detail [26]. Both viruses were propagated in rabbit kidney cells (RK13) (ATCC, Manassas, VA, USA) in MEM medium supplemented with 0.292 g/l L-glutamine, 1mM sodium pyruvate, 50μg/ml gentamycin (all Thermo Fisher Scientific Waltham, MA, USA), and 10% fetal calf serum (Atlanta biological, Flowery Branch, GA, USA).

Virus titers were determined in Ab4 and Ab4ΔORF1/71 viral stocks by virus titration (TCID50) as previously described [16, 45]. In addition, viral plaque forming units (PFU) were determined using a viral plaque assay as described below. After EHV-1 infection, TCID50 and PFU were determined again in the remaining solutions from both infection stocks to confirm the amount of virus used for infection. In addition, a PCR was performed on both infection stocks to confirm the presence of Ab4 and Ab4ΔORF1/71 in the respective stocks. PCR primers flanking the ORF1/ORF2 gene region were used: forward primer—5'- AACAACCCTGGGCTCTTTA -3' and reverse primer—5'- GATTCGCACCTCATCTCCAC -3'. Platinum Taq High Fidelity polymerase (Thermo Fisher Scientific, Waltham, MA, USA) was used for PCR amplification to generate fragments of 2043 bp and 1435 bp from genomic DNA of Ab4 and Ab4ΔORF1/71 virus stocks, respectively. Finally, the PCR products were sequenced at the Cornell Biotechnology Core Facility to confirm the integrity of the ORF1 gene in the Ab4 strain and its deletion in the Ab4ΔORF1/71. The ORF1 sequences in the virus used for experimental EHV-1 infection were 100% homologous to GenBank accession # NC_001491.2. The presence or deletion of ORF71 in Ab4 and Ab4ΔORF1/71, respectively, was confirmed by PCR as previously described [40]. The amplified ORF71 sequence from Ab4 was 100% homologous to GenBank accession # NC_001491.2.

### Experimental EHV-1 infection

EHV-1 infection was performed in November 2013 when the horses were 2.5 years of age. Horses were randomly assigned to three groups of five horses each. One group (2 mares, 3 geldings) was not infected with EHV-1 (control). A second group (2 mares, 3 geldings) was infected with the neuropathogenic EHV-1 strain Ab4. The third group (1 mare, 4 geldings) was infected with Ab4ΔORF1/71.

The horses were moved into isolation barns with individual box stalls two days prior to infection and allowed to acclimate. The stalls did not allow the horses to have direct nose-to-nose contact. The non-infected control group was housed in a separate barn. The two infected groups were housed in a barn with separate sections divided by a barrier where movement or airflow between sections was not possible. Each section had a separate entry with an area for donning and removing personal protective equipment (PPE) including disposable coveralls, boots, caps and gloves. Control horses were always handled first and people upon entering and exiting each of the sections changed PPE and applied hand sanitizer to prevent the spread of virus between the three groups. Within each section, the barns had a center hallway with the same airspace and horses were handled in each section as one group. No other specific care was taken to prevent spread of virus from horse-to-horse within each group by animal handlers with the exception of changing gloves after taking nasal swab samples from each horse.

Baseline serum and nasal secretion samples were taken the day before EHV-1 infection (d-1). Baseline physical examination measurements were taken on day -1 and immediately before EHV-1 infection (d0). Viral inoculation was performed using a mucosal atomizer device (Wolfe Tory Medical, Salt Lake City, UT). Horses in each group received one of the following preparations coded for blinding purposes: 5 ml cell culture medium without any virus; 1 x 10^7^ plaque-forming units (PFU) of EHV-1 strain Ab4 in 5 ml of medium; or 1 x 10^7^ PFU of the Ab4ΔORF1/71 virus in 5 ml of medium.

A veterinarian evaluated the general condition of the horses twice daily. The horses did not develop severe disease and interventions were not indicated at any time throughout the study. After sampling on day 16 post infection (d16pi), the horses were released from the isolation barn and kept in the experimental groups on separated pastures without contact between the different groups.

### Clinical evaluation

Each day, the control group was evaluated first, followed by the two infected groups. Due to the nature of these groupings (non-infected horses in a separate barn), the clinicians taking samples and performing the examinations were aware of the “non-infected control group” but were blinded as to which EHV-1 virus was administered to either infected group. Temperatures were taken in the morning and evening until d7pi and then once daily before samples were obtained in the mornings. A fever was defined as a rectal temperature of >38.5° C. Clinical scores for nasal discharge, eye discharge, mandibular lymph node enlargement and a neurologic score were given by a veterinarian and summed to determine a total clinical score for each horse. Gait evaluation was performed while horses were led in the aisle of the isolation barns and neurologic status was scored according to the system described by Furr and coworkers [46].

### Sample collection, processing and PBMC isolation

Blood samples (100 ml/horse/day) were collected by jugular venipuncture using a 20-gauge needle with a vacutainer system into tubes with sodium heparin for PBMC isolation or without anti-coagulant for serum collection. Blood samples were obtained on d-1, 1 to 11, 13, 16, 32, 72, 114pi. Blood in tubes without coagulant was allowed to clot at room temperature and after centrifugation (3,000xg, room temperature, 15 min) the serum was collected and frozen in aliquots at -20°C until used for quantification of EHV-1 specific antibodies and cytokines. PBMC were isolated from heparinized blood by density gradient centrifugation (Ficoll-Paque Plus, GE Health- care, Piscataway, NJ) within a few hours after blood collection and were immediately processed for virus isolation, and cellular re-stimulation experiments as described below.

Nasal swabs were obtained on the same days as the blood samples with the addition of d12 and 14pi using two sterile, polyester tipped swabs (Puritan Medical Products Company, LLC, Guilford, ME) placed in the nostril contacting the nasal mucosa for up to 2–3 seconds. The swabs containing nasal secretions were placed directly into polypropylene tubes containing 1 ml of PBS. The samples were maintained at 4°C until processing within a few hours after collection. Two nasal swab samples were taken per horse each day. One was used for virus isolation the day of collection. The second samples were frozen at -80°C and used for determination of cytokines and antibodies in the nasal secretion.

### Virus isolation in nasal secretions and PBMC

Virus isolation from nasal secretions was performed to assess viral shedding. RK13 cells were cultured in MEM medium as described above. For all viral cultures containing nasal secretions 0.75ng/l Amphotericin B (Thermo Fisher Scientific, Logan, UT) was added to the medium. Nasal swabs in PBS were vortexed in the collection tubes. Subsequently, serial dilutions of the fluid were added to the RK13 cells. Plates were incubated for 2–4 hours at 37°C in a humidified CO_2_ incubator to allow the virus to attach to the cells. Afterwards, the medium was replaced by medium containing 0.5% w/v Methylcellulose (Sigma Aldrich, St. Louis, MO) and plates were incubated for 5 days. Then, plates were washed twice with PBS, followed by fixation and staining with a crystal violet solution (PBS containing 0.05% w/v crystal violet, 4% v/v paraformaldehyde, 1% v/v methanol (all Sigma Aldrich, St. Louis, MO)) for 20 minutes at room temperature. Plates were washed with tap water and allowed to dry. They were evaluated for viral plaques by eye and by microscopy if necessary. Results were expressed as PFU per 1ml PBS solution.

Virus isolation from PBMC was performed to determine cell-associated viremia. PBMC were counted and a serial dilution ranging from 1x10^7^ to 1x10^4^ PBMC per RK13 cell well was added to the culture medium (without Methylcellulose). Plates were incubated for 5 days, fixed and stained as described above.

### Cell-associated viremia

Real-time PCR was performed using PBMC to determine cell-associated viremia as previously described [18]. In brief, DNA was isolated from aliquots of 5 x 10^6^ snap frozen PBMC using the MagMAX Total Nucleic Acid Isolation Kit (ThermoFisher). The real-time PCR reaction was set up using DNA from an equivalent of 3.42x10^5^ PBMC to determine relative viral genome levels targeting the EHV-1 gB gene [47]. The efficiency of the assay was established as 92.74% (95% CI 89.14%-96.61%) using 10-fold dilutions of infected RK13 lysates (1−10^6^ TCID50/ml). A positive cutoff was imposed at the analytic limit of detection based on 2 standard deviations above the mean Ct value for 100% of purified gB amplicon detection. Cycle threshold (Ct) values were calculated based on the automated algorithm on the Applied Biosystems 7500 platform.

### Quantification of EHV-1 specific antibodies in nasal secretion and serum

Antibodies specific for EHV-1 glycoproteins gB, gC and gD were measured by an EHV-1 multiplex assay as previously described [21]. A slight variation was performed during the fluorescent bead coupling step of the assay run here compared to the previously reported method: instead of coupling the recombinant EHV-1 glycoproteins directly to the beads, a monoclonal anti-equine IL-4 antibody (clone 25, [48]) was initially coupled to all three beads numbered 33, 35 and 36 (Luminex Corp.). Bead 33 was then incubated with IL-4-tagged EHV-1 gB, bead 35 with IL-4 tagged EHV-1 gC, and bead 36 with IL-4-tagged EHV-1 gD. All three IL-4-tagged EHV-1 glycoproteins were expressed exactly as described previously [21]. After EHV-1 gB, gC and gD incubation, the beads were washed. This step was followed by incubation of the beads with serum or nasal secretion, respectively. Serum was run at a dilution of 1:400. Nasal secretions were measured undiluted. Afterwards, detection with a polyclonal biotinylated anti-IgG(H+L) detection antibody (Jackson Immunoresearch Laboratories, West Grove, PA) followed by streptavidin-phycoerythrin (Invitrogen, Carlsbad, CA) was performed to analyze total EHV-1 gB, gC or gD specific antibodies. Serum samples were also run in additional EHV-1 multiplex assays with equine isotyping reagents including antibodies against IgG1, IgG1/3, IgG4/7, IgG3/5, IgG6 and IgM as previously described [8, 21], and anti-equine IgA (clone BVS2, [49]) for detection of EHV-1 specific IgA.

### Cellular *in vitro* re-stimulation assay

EHV-1 re-stimulation of PBMC was performed as previously described in detail [8, 18, 21]. In brief, PBMC were cultured in cell culture medium (Dulbecco's Modified Eagle Medium (DMEM) (Gibco, Invitrogen, Grand Island, NY) containing 10% (v/v) FCS (Thermo Scientific, Logan, UT), 1% (v/v) non-essential amino acids, 2 mM l-glutamine, 50 mM 2-mercaptoethanol, 50 mg/ml gentamicin). Cells were either kept in cell culture medium alone, were infected with EHV-1 strain Ab4 at a multiplicity of infection (MOI) of 1, or stimulated with phorbol 12-myristate 13-acetate (PMA; 25 ng/ml) and ionomycin (1 μM; both Sigma, St. Louis, MO). The PMA/ionomycin stimulation was used as a positive and viability control of the PBMC, and high cytokine values were measured after PMA/ionomycin stimulation for all samples and cytokines described. PBMC were cultured for 48 hours in a 5% CO_2_ incubator at 37°C. For intracellular staining of cytokines and flow cytometric analysis, the secretion of proteins from the cells was blocked by adding Brefeldin A (10 μg/ml; Sigma, St. Louis, MO) to the plates after 24 hours of culturing. The supernatants from cultures without Brefeldin A were collected after 48 hours for cytokine quantification.

### Flow cytometric analysis of EHV-1-specific T-cells

Tri-color staining and flow cytometric evaluation of the cells was performed as previously described [18, 50, 51]. In brief, following culture for 48 hours, re-stimulated cells were collected, fixed, and permeabilized. Cells were divided in two aliquots and triple stained for either CD4 and CD8 on the cell surface and intracellular IFN-γ production, or intracellular IL-10, IL-4, and IL-17A production using monoclonal antibodies. The antibodies were directly labeled and isotype-matched staining controls were used. The cells were analyzed in a FACS Canto II flow cytometer (BD Biosciences, San Diego, CA). Evaluation of the data was performed using FlowJo software version 10.2 (FlowJo LLC, Ashland, OR, USA). An analysis gate was set on the small lymphocyte population based on cell morphology and cells in this gate were then analyzed for cytokine production as previously described [21]. The percentages of EHV-1-specific IFN-γ positive lymphocytes after re-stimulation were compared to medium controls and were evaluated as previously described [8, 18, 21].

### Cytokine detection by multiplex assay

Cell culture supernatants from EHV-1 re-stimulation assays of PBMC were evaluated using a fluorescent bead-based Equine Cytokine Multiplex assay detecting IFN-α, IL-4, IL-10, IL-17 and IFN-γ as previously described [52]. The Equine Cytokine Multiplex assay is available through the Animal Health Diagnostic Center at Cornell University. IL-4, IL-10 and IFN-α were reported in pg/ml and IL-17 and IFN-γ were reported as U/ml.

In addition, cytokines and soluble CD14 (sCD14) in serum and nasal secretion samples were measured using the Equine Cytokine Multiplex assay and a sCD14 assay [53]. Serum sCD14 was reported in ng/ml.

### Statistical analysis

D’Agostino & Pearson normality tests indicated that values on most days were not normally distributed. All clinical, viral, humoral and cellular immune parameters were compared by repeated-measures ANOVAs with Tukey’s post tests for multiple comparisons between the three groups. P-values of <0.05 were considered significant. The statistical analysis was performed and the graphs were created using GraphPad Prism 6 for Mac OS X, version 6f.

## Results

### Body temperatures and clinical signs after experimental infection with the deletion mutant Ab4ΔORF1/71 and the parent Ab4 virus

**Fig 1:**
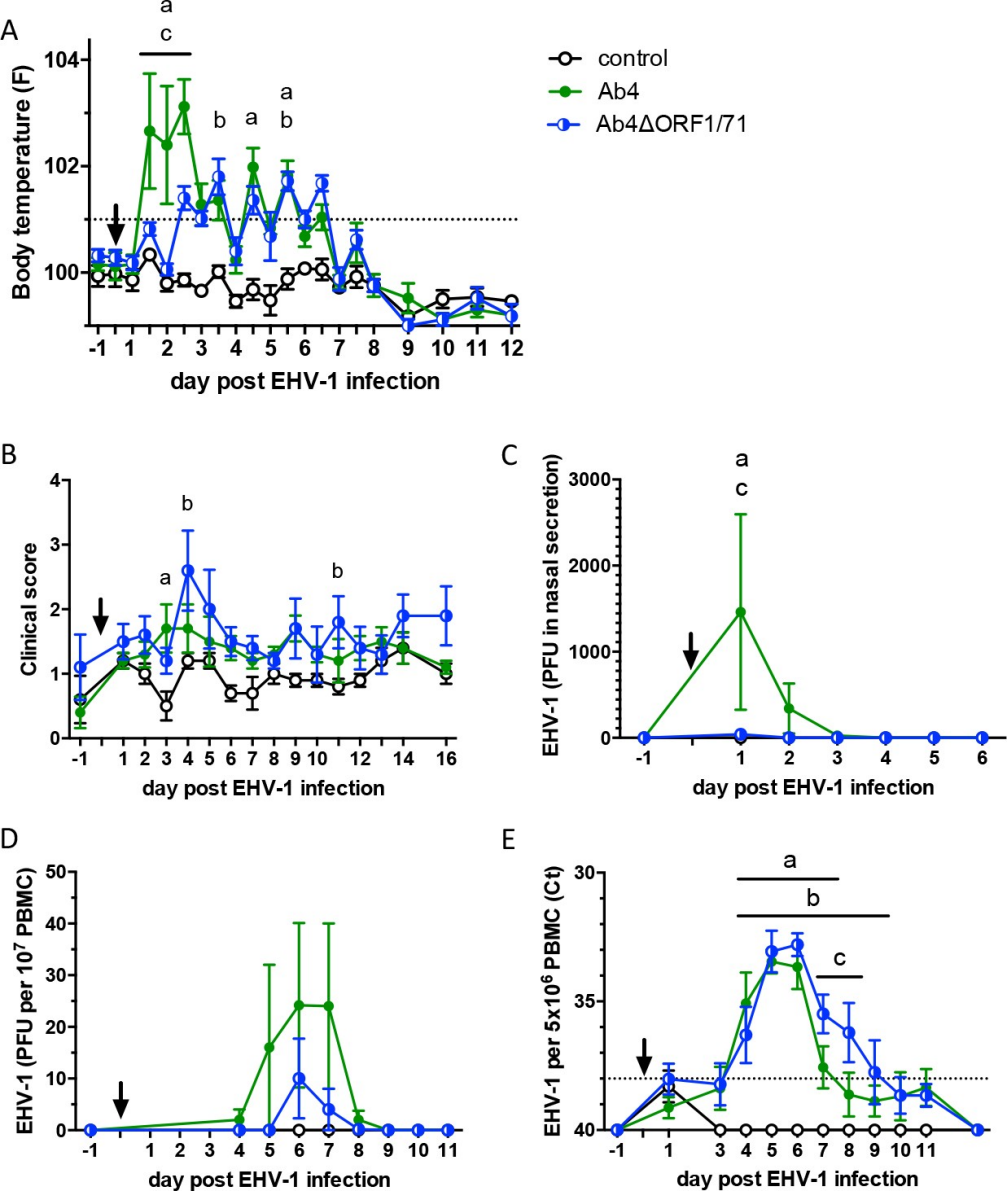
Body temperatures, clinical scores, nasal viral shedding and viremia after experimental EHV-1 infection of naïve horses. The horses were infected with EHV-1 strain Ab4 (n = 5) or deletion mutant strain Ab4ΔORF1/71 (n = 5). The arrow points to the time of infection (d0). A non-infected control group (n = 5) was included. (A) Body temperatures. The dotted line shows the cut-off value for fever of 38.5°C; (B) total clinical score determined by the summation of several clinical variables evaluated on a numerical scale; (C) EHV-1 in nasal secretions measured by virus isolation; (D) viremia measured by virus isolation from PBMC; (E) viremia determined by real-time PCR. The dotted horizontal line shows the positive PCR Ct-value cut-off value. All graphs show means and standard errors by group. Significant differences between groups are marked: a = Ab4 vs. controls, b = Ab4ΔORF1/71 vs. controls, and c = Ab4 vs. Ab4ΔORF1/71.

Horses infected with the Ab4 virus showed an initial high fever peak between 36 to 60 hours pi (d1.5, d2, and 2.5pi) with significantly higher body temperatures than non-infected controls (all p<0.0001). Horses infected with Ab4ΔORF1/71 did not show this initial fever characteristic for EHV-1 infection and had lower body temperatures than the Ab4 group with p<0.05, 0.0001, and 0.05, on days 1.5, 2 and 2.5 pi, respectively (Fig 1A). In fact, horses in the Ab4ΔORF1/71 group did not show a fever until day 2.5 pi. Body temperatures between the Ab4ΔORF1/71 and control groups were not different until d3pi. After d3pi, horses in the Ab4 and Ab4ΔORF1/71 groups showed a low-grade fever in the evenings of d3.5–6.5pi. Compared to the control group, the body temperature of the Ab4 group was increased on d4.5 (p<0.0001) and d5.5 (p<0.01), while the Ab4ΔORF1/71 groups had elevated temperatures on d3.5 and d5.5 (both p<0.05).

Infected horses showed clinical signs of mild respiratory disease. None of the horses developed neurological signs. The total clinical score, which evaluated nasal discharge, ocular discharge, lymph node enlargement, and neurological signs, was similar between the Ab4 and Ab4ΔORF1/71 groups at most time points during the study (Fig 1B). Higher clinical scores than in non-infected controls were only observed on d3pi in the Ab4 group (p<0.01) and on d4 and 11pi the Ab4ΔORF1/71 group (p<0.01 and p<0.05, respectively).

### Viral shedding and viremia

EHV-1 was not isolated from nasal secretions of horses in the non-infected group. In the Ab4 group, EHV-1 was isolated from nasal secretions of all horses on d1pi, of three horses each on d2 and d3pi, and of one horse on d4pi (S1 Table). Only 1 out of 5 horses infected with the Ab4ΔORF1/71 deletion mutant virus had virus present in nasal secretions on d1-2pi. Overall, nasal shedding in Ab4 infected horses was significantly increased compared to the other two groups (both p<0.05) on d1pi (Fig 1C). Virus isolation was performed daily until d9, and also on d11 and d13pi with no virus isolated beyond d4pi.

EHV-1 viremia was evaluated in PBMC by virus isolation (Fig 1D) and also by PCR (Fig 1E). EHV-1 was not isolated from or detected in PBMC of the control horses. All horses in both infected groups developed viremia. Virus isolation identified infectious virus in PBMC from horses infected with Ab4 or Ab4ΔORF1/71 between d4-8pi with peak amounts of infectious virus on d6pi. Viral DNA was detected by PCR in PBMC of Ab4 infected horses and was increased compared to the control group on d4-6pi (all p<0.0001) and d7pi (p<0.05). Ab4ΔORF1/71 infected horses had viral DNA in their PBMC from d4-9pi (Fig 1E) which was significantly higher than in control horses. On d7 and 8pi, Ab4ΔORF1/71 infected horses had increased viral DNA copies in their PBMC compared to the Ab4 group (both p<0.05).

### Intranasal immune response after EHV-1 infection

**Fig 2:**
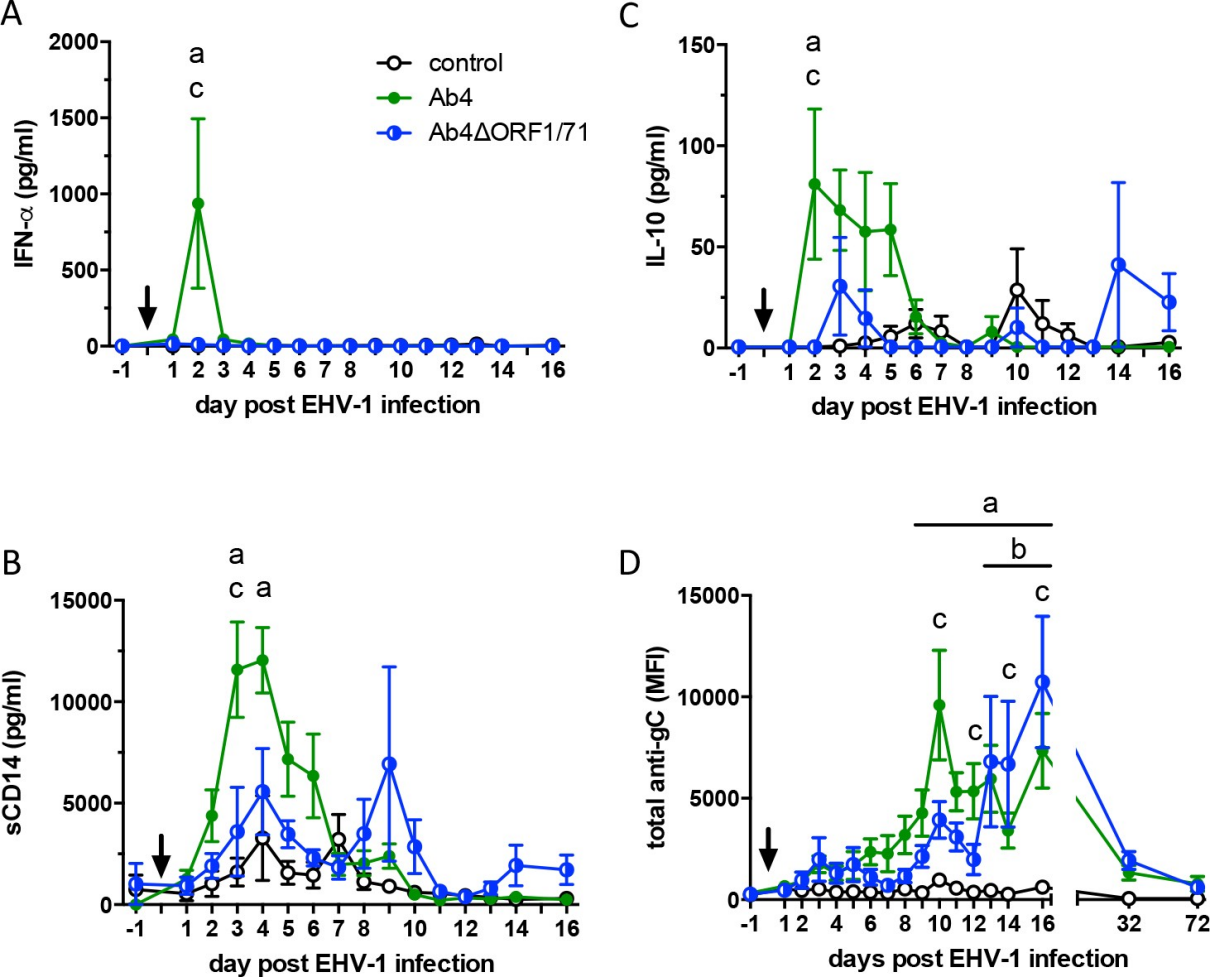
Cytokines and total anti-EHV-1 gC antibodies in nasal secretions of naïve horses (n = 5 per group) after infection with EHV-1 strain Ab4 or its deletion mutant Ab4ΔORF1/71. Non-infected horses were kept as controls. The arrow marks the time of infection. Nasal secretion samples were collected before and at various times after infection. Cytokine concentrations were analyzed by a bead-based Cytokine Multiplex assay and antibodies were evaluated by EHV-1 multiplex assay: (A) IFN-α (B) sCD14, (C) IL-10, and (D) total anti-gC antibodies expressed as median fluorescence intensities (MFI). Mean and standard errors are displayed. Significant differences between groups: a = Ab4 vs. controls, b = Ab4ΔORF1/71 vs. controls, and c = Ab4 vs. Ab4ΔORF1/71.

Infection with EHV-1 Ab4 induced an intranasal cytokine response characterized by a sharp increase in IFN-α on d2pi (p<0.0001) and elevated IL-10 staring on d2pi (p<0.01) on d2pi compared to the Ab4ΔORF1/71 infected and non-infected control groups (Fig 2A and 2B). In addition, sCD14 was significantly increased in Ab4 infected horses compared to horses in the Ab4ΔORF1/71 group on d3pi (p<0.01) and compared to non-infected controls on d3pi (P<0.0001) and d4pi (p<0.001) (Fig 2C). The increase in IFN-α, IL-10 and sCD14 in the Ab4 group occurred simultaneously with the initial fever peak and nasal viral shedding in this group (Fig 1A and 1C). Intranasal IFN-α, IL-10 and sCD14 did not differ between the Ab4ΔORF1/71 infected and uninfected control groups. Intranasal IFN-γ, IL4, or IL-17 levels were overall low and did not differ between all three groups before or after infection (data not shown).

Intranasal anti-EHV-1 gC antibodies were significantly increased in the Ab4 group compared to non-infected controls between d9-16pi (Fig 2D) and peaked on d10pi. Intranasal antibodies developed more gradually after Ab4ΔORF1/71 infection than after Ab4 infection, were higher than in non-infected controls between d13-16pi (Fig 2D), and peaked on d16pi. Intranasal anti-gC antibodies were higher in Ab4 than in Ab4ΔORF1/71 infected horses on d10 (p<0.0001) and d12pi (p<0.05). However, between d14-16pi the Ab4ΔORF1/71 group demonstrated a higher antibody response then the Ab4 group (p<0.05). By d32pi the intranasal anti-gC antibody response had returned to baseline in both infected groups. Intranasal anti-gB and anti-gD antibodies were similar to the antibody response against EHV-1 gC in onset and magnitude (S1 Fig).

### EHV-1 specific antibody response in serum

**Fig 3:**
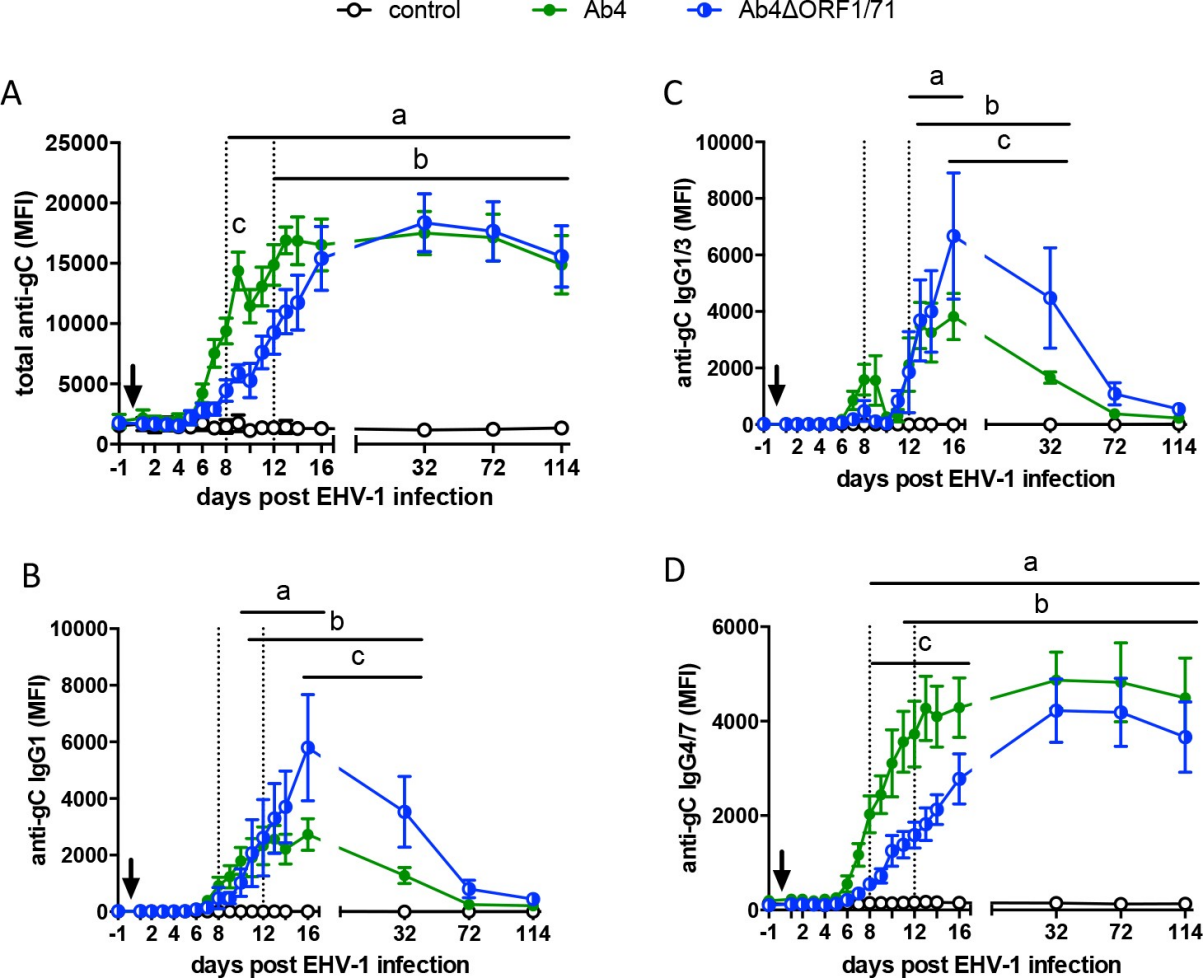
Total anti-EHV-1 gC and IgG isotype responses in serum from horses after infection with the EHV-1 strain Ab4 or its deletion mutant Ab4ΔORF1/71. EHV-1 naïve horses (n = 5 per group) were infected with the EHV-1 strain Ab4 or Ab4ΔORF1/71. Non-infected horses were kept as controls. The arrow marks the time of infection on d0. Serum antibodies were measured by an EHV-1 Multiplex assay. Antibody values are expressed as median fluorescence intensities (MFI) for (A) total Ig, (B) IgG1, (C) IgG1/3, and (D) IgG4/7. All graphs show means and standard errors by group over time. The dotted vertical lines mark d8 and d12pi to illustrate the time of the onset of the response for total Ig and the different IgG isotypes. Significant differences between the groups are shown: a = Ab4 vs. controls, b = Ab4ΔORF1/71 vs. controls, and c = Ab4 vs. Ab4ΔORF1/71.

The serum antibody responses against EHV-1 gB, gC, and gD were similar for both total antibody and isotypes measurements. Representative results for antibodies against EHV-1 gC are presented here (Fig 3).

Before infection (d-1), anti-gC EHV-1 antibodies in serum of all groups were below the positive cut-off range of the assay. In both infected groups, Ab4 and Ab4ΔORF1/71, anti-gC antibodies were induced. In these groups, antibodies were first detectable on d6pi (Ab4) or d8pi (Ab4ORF1/71) and started to plateau after d13pi (Ab4) or d16pi (Ab4ΔORF1/71) (Fig 3A). Anti-gC antibody values in both infected groups remained increased through the end of study period on d114pi. None of the control horses displayed a serological response against EHV-1 during this time. The initial increase of anti-gC antibodies was more rapid in the Ab4 infected horses than in Ab4ΔORF1/71 infected horses. Compared to the control group, the Ab4 group anti-gC antibodies increased on d8pi (p<0.01) and stayed high afterwards (d9-d114pi: all p<0.0001). In the Ab4ΔORF1/71 group, serum anti-gC antibodies were first higher than in control horses on d12pi (p<0.05) and from then on (d14pi: p<0.001; d16-d114pi: all p<0.0001). The slower onset of serum antibody development in the Ab4ΔORF1/71 group compared to the parent Ab4 virus infected group was also reflected in significantly different antibody values between these two groups on d9pi (p<0.01).

EHV-1 gC-specific IgG1 and IgG4/7 isotypes dominated the gC-specific antibody response in serum (Fig 3B and 3D). IgG4/7 antibodies overall resembled the total antibody response with a more rapid onset of anti-gC IgG4/7 in the Ab4 (d8pi) than in the Ab4ΔORF1/71 group (d11pi) and long-lasting antibodies until the end of the study (d114pi) in both infected groups (Fig 3D). In contrast, anti-gC IgG1 started to increase slightly after IgG4/7 antibodies in the Ab4 group (d10pi) and simultaneously with IgG4/7 in the Ab4ΔORF1/71 group (d11pi). After d16pi, IgG1 antibodies declined rapidly in both infected groups and were similar to the control group from d32pi (Ab4) on. Horses infected with the Ab4ΔORF1/71 deletion mutant developed higher anti-gC IgG1 responses until d32pi (Fig 3B). IgG1/3 antibody responses resembled IgG1 antibodies and were therefore considered to be composed almost exclusively of IgG1 (Fig 3C). In both infected groups, gC specific IgG3/5 was induced at low levels and was not increased compared to the control group at any time (S2B Fig). In addition, anti-gC IgG6 antibodies were induced at very low values after infection. The IgG6 values increased on d13 to 32pi (Ab4ΔORF1/71) or d16pi (Ab4) compared to the controls, and declined after d16pi in both infected groups (S2C Fig).

EHV-1 infection did not induce a major serum gC-specific IgM response during the acute phase of the infection and IgM antibodies remained low in all groups throughout the study period (S2A Fig). However, serum IgM values in the control group were elevated on d72 (p< 0.01) and d114pi (p<0.001) compared to the Ab4ΔORF1/71 infected group This is likely an artificial difference resulting from higher levels of anti-gC IgG in the sera of the infected groups combined with some minor non-specific IgM reactivity in the control group samples. Anti-gC IgA was not detected in serum at any time (data not shown).

### Characterization of cellular immune responses

**Fig 4:**
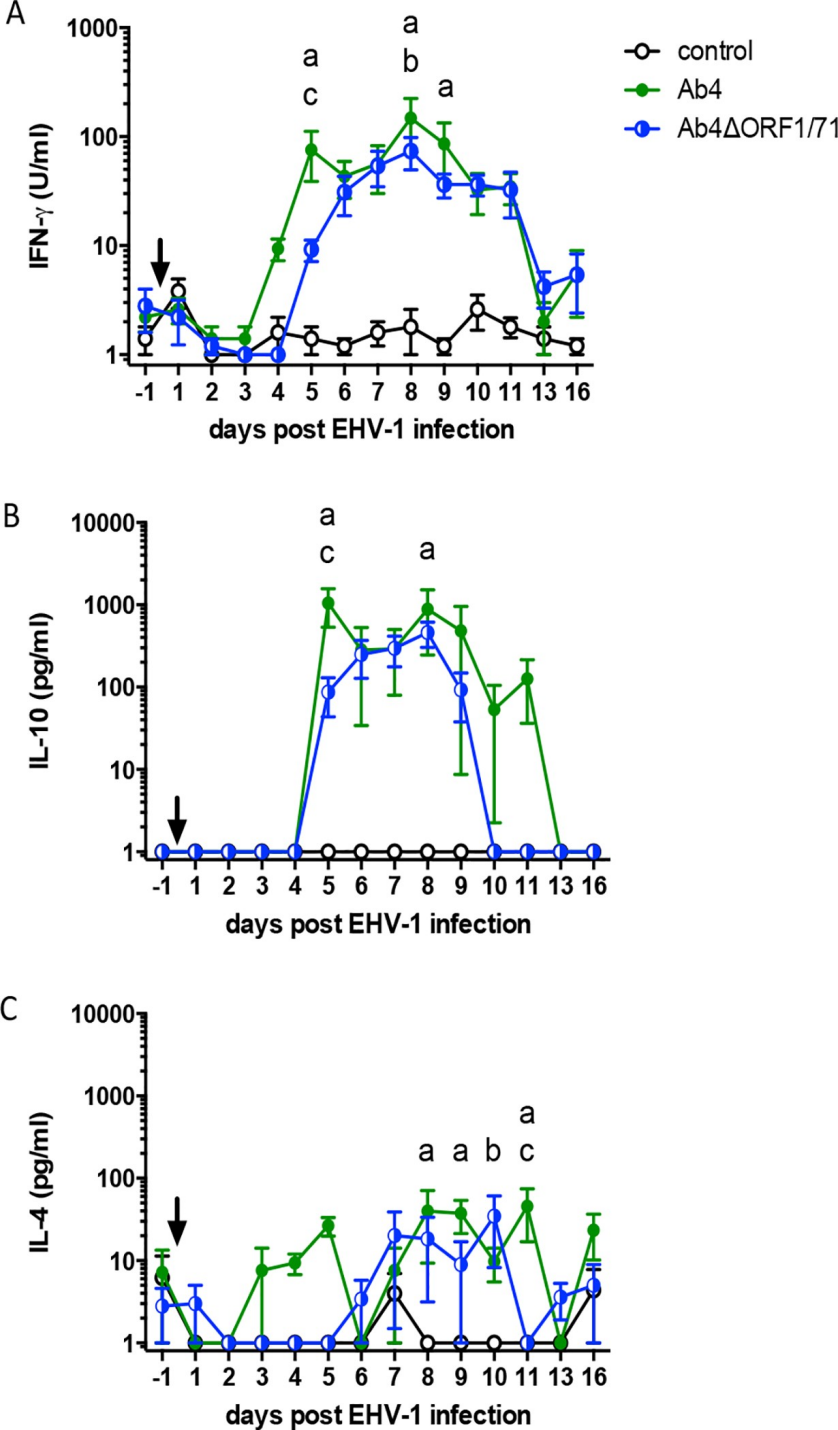
Cytokine secretion from PBMC of EHV-1 infected horses after re-stimulation *ex vivo*. EHV-1 naïve horses were infected with one of two EHV-1 strains (Ab4 or Ab4ΔORF1/71). A group of non-infected control horses was included (n = 5 per group). The arrow points to the time of infection. PBMC were harvested before and at several time points after infection and were re-stimulated with the EHV-1 strain Ab4 or were kept in cell culture medium. After 48 hours of incubation cell culture supernatants were harvested and cytokine production was analyzed by a bead-based Cytokine Multiplex assay. All EHV-1 re-stimulation values shown in the figures are corrected by the value of the respective medium control. Mean and standard errors for (A) IFN-γ, (B) IL-10, and (C) IL-4 in the cell culture supernatants are displayed. Significant differences between groups: a = Ab4 vs. controls, b = Ab4ΔORF1/71 vs. controls, and c = Ab4 vs. Ab4ΔORF1/71.

Cellular immune responses following infection were compared between the three groups after re-stimulation of PBMC with EHV-1 Ab4 *ex vivo*. Secreted cytokines were measured in the supernatants. Overall, IFN-γ and IL-10 secretion from EHV-1 re-stimulated PBMC was detectable in the infected groups within two weeks pi while PBMC from non-infected horses did not secrete these two cytokines (Fig 4A and 4B). Specifically, IFN-γ secretion started to rise in the Ab4 group by d4pi and was increased on d5 (p<0.01), d8 (p<0.0001) and d9pi (p<0.01) compared to the control group. In the Ab4ΔORF1/71 group, IFN-γ production became detectable on d5pi and was increased compared to non-infected controls on d8pi (p<0.01). PBMC from horses infected with Ab4 had higher IFN-γ production compared to those infected with Ab4ΔORF1/71 on d5 (p<0.05) (Fig 4A). IL-10 secretion from PBMC of horses infected with Ab4 was increased on d5 (p<0.0001) and d8pi (p<0.001) compared to non-infected controls. On d5pi, the IL-10 concentration in the Ab4 group was also higher than in the Ab4ΔORF1/71 group (p<0.0001). Between d6-9pi, mean IL-10 concentrations were similar between the two infected groups, even if the Ab4ΔORF1/71 group IL-10 concentrations did not reach significance compared to the non-infected group (Fig 4B). Beyond d16 pi, IFN-γ and IL-10 secretion from EHV-1 re-stimulated PBMC was no longer detectable or only detectable for single horses at low values on d72 pi (data not shown). IL-4 was induced at low concentrations in cells from infected horses and mostly in the second week pi (Fig 4C). In comparison to the non-infected control group, IL-4 secretion was elevated in the Ab4 group on d8 (both p<0.05), and d11pi (p<0.01), while IL-4 secretion in the Ab4ΔORF1/71 horses was increased on d10pi (p<0.05). On d11pi, IL-4 secretion was also higher in the Ab4 group than in the Ab4ΔORF1/71 group (Fig 4C). IL-17 was not detected and IFN-α was continuously secreted pre- and post-infection (d-1 to d16pi) by EHV-1 re-stimulated PBMC at high concentrations but without differences between all three groups.

**Fig 5:**
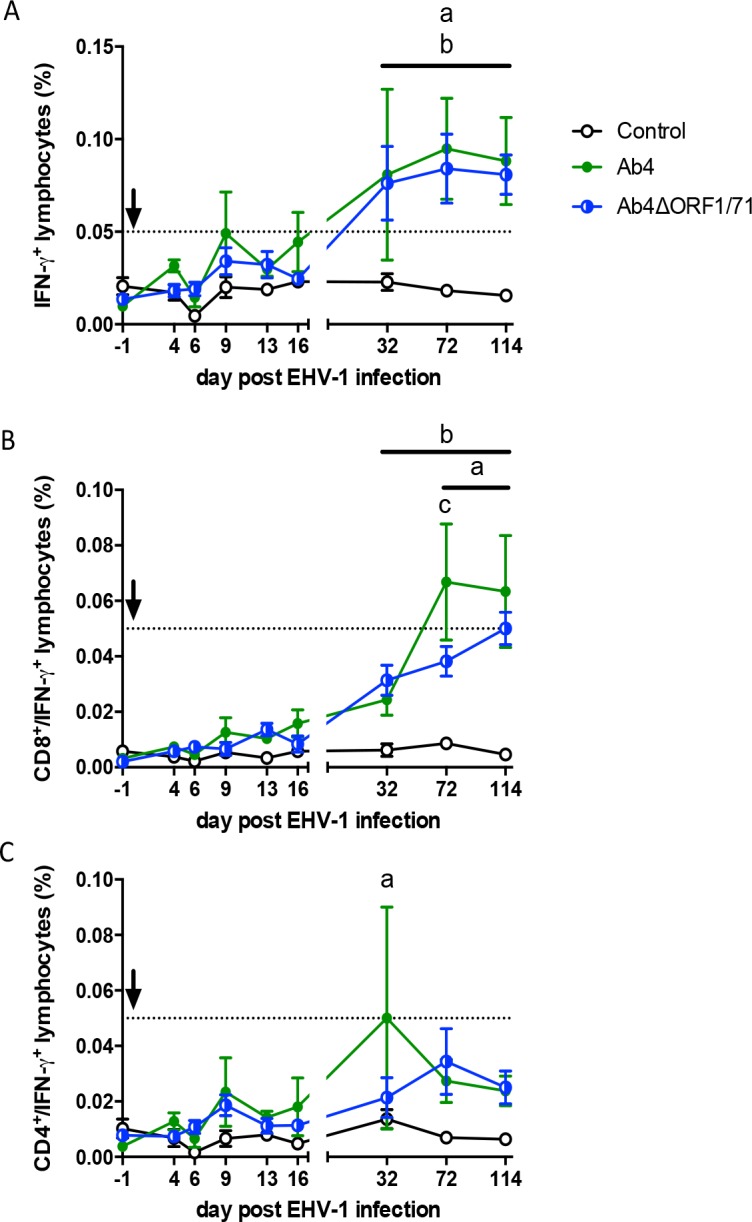
EHV-1 specific IFN-γ producing lymphocytes in PBMC. EHV-1 naïve horses (n = 5 per group) were infected with the EHV-1 strain Ab4 or the deletion mutant virus Ab4ΔORF1/71. Non-infected horses were kept as controls. The arrow marks the time of infection. PBMC were isolated at different times post infection and re-stimulated with EHV-1 strain Ab4 *ex vivo* or were kept in cell culture medium for control. The secretion inhibitor Brefeldin A was added to the cultures for the last 24 hours. Cells were harvested at 48 hours, fixed, stained for intracellular IFN-γ production and cell surface CD4 and CD8, and measured by flow cytometry. The analyzing gate was set on small lymphocytes. Mean and standard errors for the percentages of (A) total IFN-γ^+^ lymphocytes, (B) CD8^+^/IFN-γ^+^ lymphocytes, and (C) CD4^+^/IFN-γ^+^ lymphocytes are displayed over time. All percentages for IFN-γ^+^ cells were corrected by the respective medium control values. The dotted horizontal line represents a suggested cutoff of 0.05% IFN-γ^+^ lymphocytes. Values below this cutoff were typically reached for IFN-γ^+^ cells in the non-infected control group prior to correction by medium control values. Significant differences between groups are marked: a = Ab4 vs. controls, b = Ab4ΔORF1/71 vs. controls, and c = Ab4 vs. Ab4ΔORF1/71.

The cellular sources of IFN-γ, IL-10 and IL-4 were analyzed by flow cytometric analysis of EHV-1 re-stimulated PBMC. EHV-1 specific T-cells were identified by triple staining with the cell surface markers CD4 and CD8 and intracellular staining with anti-IFN-γ. IFN-γ producing lymphocytes were not detectable for all three groups pre-infection and until d16pi. Starting on d32pi, EHV-1-specific IFN-γ producing lymphocytes were increased in PBMC from both infected groups (p<0.05) compared to non-infected controls and this continued until d114pi (p<0.01) (Fig 5A). However, the percentages of EHV-1-specific IFN-γ^+^ lymphocytes were overall low with maximal percentages of around 0.1% IFN-γ^+^ cells in the Ab4 and Ab4ΔORF1/71 groups. Within these, CD8^+^/IFN-γ^+^ cells dominated the EHV-1 specific T-cell response from d32pi on (Fig 5B), while CD4^+^/IFN-γ^+^ cells remained at very low percentages throughout the study (Fig 5C). The only exception was a moderate one-time increase of CD4^+^/IFN-γ^+^ on d32pi in horses infected with Ab4. By d72pi, the CD8^+^/IFN-γ^+^ phenotype represented the majority of the IFN-γ producing lymphocytes until d114pi (Fig 5B and 5C). PBMC were also stained for IL-4, IL-10 or IL-17 which were not detected in EHV-1 re-stimulated PBMC.

### Cytokine response in serum

Before and following experimental infection different serum cytokines and sCD14 were quantified in fluorescent bead-based assays (S3 Fig). Serum sCD14 increased in the serum of both infected groups when compared to non-infected controls starting on d4pi and continuing until d6pi (Ab4) or d7pi (Ab4ΔORF1/71). Serum sCD14 was also higher in the Ab4ΔORF1/71 infected group compared to the Ab4 infected group at d16pi (S3A Fig). Serum IFN-γ was not different between groups at any point during the study. However, there was a notable increase in serum IFN-γ values compared to the pre-infection values after the first week of stabling in all groups including the non-infected controls (S3B Fig). IFN-α, IL-4, IL-10, and IL-17 were inconsistently detectable in sample from individual horses and values were overall low and without any differences between groups.

## Discussion

Previously, ORF1 was evaluated after infecting cells with Ab4ΔORF1 deletion mutants *in vitro*. In comparison with its parent Ab4 strain, it was identified that the ORF1 gene contributed to down-regulation of MHC-I, modulation of IFN-α and IL-10 expression, and down-regulation of chemokine expression limiting chemotaxis of monocytes and neutrophils, thereby aiding in immune evasion [26, 27]. ORF71 was found to play a role in immune modulation and respiratory pathology of EHV-1 in mice [54]. The ORF1/71 gene deletion [26] was developed with the expectation of limiting immune evasion and virulence with the resulting strain combined with more effective immunity and longer lasting memory to protect against the severe clinical manifestations of EHV-1 infection.

In this study, we have explored the influence of the ORF1 and ORF71 genes of EHV-1 on clinical disease, nasal shedding, viremia and immune induction. The gene deletion mutant virus, Ab4ΔORF1/71, appeared to be a valid vaccine candidate based on its ability to induce antibody responses and cellular immunity similar to the parent neuropathogenic Ab4 strain while being less virulent as it induced less fever and reduced nasal viral shedding.

Biphasic pyrexia has been observed during wild type EHV-1 infection [55] and was also seen here in the Ab4 group. The absence of the initial fever spike in the Ab4ΔORF1/71 infected group demonstrates the lower virulence of this virus. Overall, mild respiratory signs were observed in both infected groups, while neurological signs were not induced. The absence of neurologic signs is not entirely unexpected as other experimental EHV-1 infection studies using neuropathogenic strains such as Ab4 resulted in 0–35% of horses demonstrating neurologic signs [16, 25]. Similarly, 3–45% of horses have been reported to be affected by neurologic signs in natural outbreaks in the US and Europe [10, 56, 57, 58]. Young mature horses were used in this study. In one previous study, a positive correlation was observed between aged mares (> 20 years) and development of neurologic signs with 67% developing neurologic signs following experimental infection [17]. Although increased age might be a risk factor for EHM, neurological outbreaks occur in horses of all ages [1, 16].

Although the virulence of Ab4ΔORF1/71 was reduced, the mutant virus induced viremia after infection similar to that observed for the Ab4 strain. Cell-associated viremia is considered a main contributor to the spread of EHV-1 to endothelial cells and is thereby a pre-requisite for the development of EHM and abortion [16, 59, 60]. It is thus possible that the Ab4ΔORF1/71 virus will still be able to induce neurologic disease. It is still unknown how viremia will be affected in Ab4ΔORF1/71 infected horses upon EHV1 challenge infection. This needs to be determined before further evaluating Ab4ΔORF1/71 as a vaccine candidate. It will likely be necessary to further attenuate the Ab4ΔORF1/71 strain to limit viremia after initial infection. Our finding that ORF1/71 deletion did not influence viremia was consistent with a previous experimental infection study using a double ORF1/ORF2 deletion mutant of Ab4 [25]. Together, these observations suggest that the ORF1 gene is not or only randomly responsible for the absence of viremia observed after infection with the 6-gene deletion mutant KyA, one of which is ORF1 [23].

Nose-to-nose transmission or transmission via infected fomites are the most common routes of infection for EHV-1 [1]. Here, viral shedding was clearly reduced by both duration and magnitude in the Ab4ΔORF1/71 infected group compared to Ab4. Importantly, after Ab4ΔORF1/71 infection most (4/5) horses did not shed detectable amounts of virus and the fifth horse shed virus at a low titer on only one day pi. In contrast, nasal shedding was detected in all five horses in the Ab4 infected group. These findings are consistent with studies of other EHV-1 mutants also missing the ORF1 gene [23, 25]. This suggests that the ORF1 gene of EHV-1 is involved in viral replication in the equine nasal epithelium.

In agreement with the almost absent nasal shedding, horses infected with Ab4ΔORF1/71 did not develop intranasal IFN-α, IL-10 or sCD14 responses, while Ab4 infected horses secreted IFN-α and IL-10 at the local infection site starting on d2pi and sCD14 on d3pi. Intranasal secretion of these three markers occurred simultaneously with the initial fever spike and viral shedding. IFN-α is known for its anti-viral activity *in vitro* alarming neighboring cells and the immune system about the invader [61, 62, 63]. However, during *in vivo* infection with EHV-1 IFN-α rather represents a ‘danger signal’ that seems to be linked to the amount of virus in the nasal secretion. The absence of detectable intranasal IFN-α together with reduced anti-inflammatory IL-10 and inflammatory sCD14 production compared to the parent Ab4 strain points to an overall decreased mucosal innate immune response as a consequence of the ORF1/71 gene deletions from Ab4. This further supports the reduced virulence of the Ab4ΔORF1/71 virus. Despite the reduced innate immune induction at the site of infection, the intranasal antibody response was not compromised by the ORF1/71 deletion. Although the intranasal anti-gC response started slower after Ab4ΔORF1/71 infection, it was compensated by a higher magnitude than the anti-gC response induced by Ab4 on days 14 and 16pi. The slower onset of the intranasal antibody response against Ab4ΔORF1/71 is likely also a result of the lower amount of virus at the local infection site.

Homologs of ORF1, genes of the UL56 family, in other alphaherpesviruses encode proteins similar to that in EHV-1 [64]. The homologous proteins of herpes simplex virus (HSV-)1 and HSV-2 are dispensable for virus growth *in vitro* but contribute to pathogenicity in mice [65, 66]. Similarly, we observed reduced virulence after infecting horses with Ab4ΔORF1/71 compared to Ab4. This, together with the evidence from HSV models, indicates that ORF1 is a virulence factor of alphaherpesviruses and its deletion is likely beneficial for attenuation during vaccine development.

The induction of serum antibodies against both viruses, Ab4 and Ab4ΔORF1/71 was similar, again with the exception of the slightly slower onset of the systemic antibody response after Ab4ΔORF1/71 infection. It can be concluded that the deletion of the ORF1 and ORF71 genes from the Ab4 virus did not influence the magnitude or duration of the antibody response after infection. The slightly delayed onset of the serum antibody response in horses infected with Ab4ΔORF1/71 may again be a consequence of the lower intranasal virus load as a result of reduced replication at the infection site, similar to local antibodies at the nasal mucosa discussed above. Serum antibody responses to both viruses were dominated by IgG1 and IgG4/7. IgG4/7 closely mimicked total anti-gC antibodies over the whole period of observation. IgG1 antibodies were induced in a short-term response declining after 2 months, while IgG4/7 antibodies sustained for at least 4 months pi. An IgG response dominated by IgG1 and IgG4/7 isotypes is characteristic for EHV-1 infection and has been observed for various EHV-1 strains [8, 18, 25]. The overall isotype pattern of the antibody response seen here for both viruses was also similar to that seen following vaccination with an inactivated vaccine in EHV-1 naïve pregnant mares using a similar antibody assay for the measurement as used here [21]. However, some EHV vaccines induce a mixed or IgG3/5 dominated response when isotype responses were evaluated by ELISA [16, 20]. A broad IgG response dominated by IgG4/7 is believed to be protective [16, 18]. IgG1, IgG4, and IgG7 isotypes of the horse have been linked to Th1 responses, and therefore, the isotype pattern seen here indicates a Th1/IFN-γ driven B-cell response [21, 67, 68, 69].

It is notable that these EHV-1 naïve horses did not mount an obvious IgM response but instead rapidly increased IgG antibodies starting at d8pi. This suggests an initial IgG B-cell response in these EHV-1 naïve horses consisted mainly of IgG1 and IgG4/7. A similar initial IgG1 response, subsequently followed by IgG4/7, was also seen following each vaccination in a multiple vaccination study of EHV-1 naïve mares [21] and after experimental infection of EHV-1 naïve weanlings who only mounted a weak and flat IgM response similar to the IgM antibodies observed in this current study [8]. These findings suggest that anti-EHV-1 B-cell responses, even in horses that have not been exposed to EHV-1 previously, are rarely completely undetectable. One possible reason for the missing anti-EHV-1 IgM response in EHV-1 naïve horses could be that EHV-4 is endemic in Iceland and consequently the horses used in these studies have likely been exposed to EHV-4 during their lifetime. We have observed that pre-existing EHV-4 antibodies against the gC and gD antigens, which are highly homologous between EHV-1 and EHV-4, partially cross-react in the EHV-1 multiplex assay used here (data not shown). Thus, the low pre-infection EHV-1 gC and gD antibody values concurrently indicate that EHV-4 antibodies were also very low in these horses immediately prior to infection. Nevertheless, an existing memory B-cell response against EHV-4 from an exposure to EHV-4 earlier in life could have inhibited the classical onset of an initial IgM response after the experimental EHV-1 infection performed here. An alternative hypothesis for the weak IgM isotype induction observed here is that IgG, and in particular IgG1, can be induced early following EHV-1 infection in horses without a preceding high magnitude IgM response [21, 69]. The latter hypothesis is supported by the early onset and short-term longevity of IgG1 antibodies observed in this and previous experimental EHV-1 infection and vaccination studies [8, 21] and by the functional similarity between equine IgM and IgG1 which can both effectively activate complement and bin to Fc-receptors [67, 69].

Notably, the robust systemic antibody response developed quickly after infection and in the absence of a detectable systemic EHV-1 specific Th cell response, as evidenced by the lack of EHV-1-specific CD4^+^ cells in re-stimulated PBMC during the first 2–3 weeks pi. T-cell responses were first detectable one month pi, were dominated by CD8^+^ cells, and stayed at low frequencies until three months pi. The same discrepancy between detectable antibody and T-cell responses has previously been observed after infection of weanlings with the EHV-1 strain NY03 [8]. While the low EHV-1 specific T-cell response in the seven months old weanlings [8] could possibly be explained by a partially immature immune system, the T-cell responses after Ab4 infection of the 2.5 years old, fully immunologically mature horses used here was almost identical. This makes the influence of horse age or viral strain on the weak T-cell response rather unlikely. With respect to the robust antibody response, the location and mechanism of B-cell induction after EHV-1 infection is yet to be fully unraveled. Likely B-cell induction and early EHV-1-specific antibody development happens in the regional lymph nodes and lymphatic tissues of the upper respiratory tract, as mandibular lymph node swelling was noted around the time when antibodies started to appear in nasal secretion and serum. One likely possibility is that a mild, localized Th1-cell response is sufficient to induce EHV-1 specific B-cell immunity, and this response is not detectable in the peripheral blood by the assays used here. A second reason for the low EHV-1 specific T-cell response could be that EHV-1 actively down-regulates T-cell induction, leading to the delayed and weak T-cell responses in PBMC observed here and previously [8]. *In vitro*, the deletion of ORF1 improves MHC-I and cytokine expression after EHV-1 infection [26]. Although the ORF1 deletion could potentially lead to improved antigen presentation and T-cell stimulation *in vivo*, our current results showed no effect of the ORF1 deletion on circulating EHV-1 specific T-cell numbers and do thus not support the hypothesis that the ORF1 gene product inhibits T-cell immunity.

This study also clearly showed that serum cytokines are not indicative of the horse’s cellular immune response to EHV-1. Overall and in contrast to intranasal cytokine responses discussed above, serum cytokines appear to be of low diagnostic value. However, the profiles of secreted cytokines induced from PBMC after re-stimulation with EHV-1 provide some evidence for the potential of EHV-1 to interfere with T-cell induction. Secreted IFN-γ and IL-10 production from PBMC started on day 4-5pi in the absence of a detectable T-cell response in the same PBMC samples. Thus, these cytokines are likely produced by innate immune cells, such as natural killer cells or γ /∂ T-cells for IFN-γ [70, 71, 72, 73] and monocytes or plasmacytoid dendritic cells may produce IL-10 [74, 75]. IFN-γ is a potent positive driver of cellular immune responses and plays an important role in adaptive immune induction, while IL-10 is important immune regulator with anti-inflammatory potential, that may modulate the T-cell response [75, 76, 77]. After EHV-1 infection with Ab4 or Ab4ΔORF1/71, induction of IFN-γ and IL-10 secretion from PBMC was maintained during the first 11-14dpi. Afterwards, IFN-γ and IL-10 secretion from PBMC was no longer detectable or only detectable for single horses at low values until the end of the study period. This was an unexpected finding. It was expected that at least the IFN-γ values would increase and be maintained with EHV-1 specific T-cells developing. Instead, the IFN-γ secretion decreased again in PBMC isolated from horses infected two weeks pi. However, this observation was similar to results seen in the weanlings discussed above following experimental EHV-1 infection. More dramatically, in weanlings IFN-γ and IL-10 secretion from EHV-1 re-stimulated PBMC sharply increased at the end of the first week pi and then declined rapidly to undetectable values after 2 (IFN-γ) to 4 days (IL-10) [8]. Overall, these observations suggest that EHV-1 interferes with the innate immune system’s ability to produce IFN-γ, possibly by induction of regulatory IL-10 and other anti-inflammatory or regulatory cytokines. The EHV-1 interference likely resulted in the observed shut-down of IFN-γ production by PBMC which became unresponsive to re-stimulation with EHV-1. It can be hypothesized that the interference of EHV-1 with innate IFN-γ production is partially responsible for the delayed and weak EHV-1-specific T-cell induction and memory response observed after experimental infection. This hypothesis requires additional future experimental proof.

As mentioned above, adaptive T-cell immunity, measured as IFN-γ production by T-cells, was low overall and of slow onset, undetected until one-month pi. The main source of the IFN-γ from T-cells was from the CD8^+^ T-cells and was similar in both infected groups. EHV-1 stimulated IFN-γ production by T lymphocytes was previously described to be dominated by CD8^+^ cells for several months following infection [74, 75, 18], but changes over time to a dominant CD4^+^/IFN-γ^+^ response in survivors of experimental and natural infections [18, 78, 79]. The overall low T-cell induction seen here supports the above hypothesis of EHV-1 interfering with T-cell induction by down-regulating innate and adaptive cellular immune responses, suppressing cytokine production, and modulating the T-cell response. Cytotoxic T lymphocytes (CTL) are currently considered protective against EHM [2, 12, 15, 17, 59, 60, 79, 80]. This is based on the hypothesis that the development of EHM and abortion rely on viremia using the infected peripheral blood cell as a vehicle to transport EHV-1 to the endothelial cells in the CNS or uterus [2, 6, 14, 15, 16, 59, 60]. It has also been concluded that cell-associated viremia cannot be cleared by serum antibodies. Thus cell-mediated immunity in the form of EHV-1-specific CD8^+^ CTL is believed to be necessary to remove infected cells from the circulation, thereby limiting viremia and the possibility of spreading EHV-1 to endothelial cells. It has been shown experimentally that EHV-1-specific CTL protect against EHV-1 induced abortion [60], and that high amounts of EHV-1-specific CTL precursors correlated with protection against EHM in aged mares [17]. Despite all these advances in understanding host immunity against EHV-1, it continues to be important to further define responses that represent protection from EHV-1 and its more severe clinical manifestations.

## Conclusion

The results demonstrate that Ab4ΔORF1/71 is an interesting vaccine candidate because of its ability to induce antibody responses and cellular immunity similar to the parent Ab4 strain while being less virulent. Notably, Ab4ΔORF1/71 produces only a mild fever and significantly less viral shedding (80% fewer horses). Accordingly, ORF1/71 deletion reduces virulence. Viremia is not influenced by the ORF1/71 gene deletions. Further evaluation of Ab4ΔORF1/71 in a challenge and protection study is needed to determine its full potential as a vaccine candidate, especially in respect to preventing cell-associated viremia after EHV-1 challenge. Currently it is believed that a combination of humoral and cell-mediated immunity is necessary to protect from these severe clinical signs and reduced viral shedding [12, 13]. Decreasing nasal shedding prevents the spread of the virus and disease between individual horses. Reducing viremia is believed to be mechanistically required for preventing the more severe clinical manifestations of EHM and abortion [2, 6, 14, 15, 16]. We have shown here and previously [8] that the induction of cellular immunity against EHV-1 is slow and EHV-1 specific T-cell memory is weak after experimental infection. Simultaneously, strong antibody responses against EHV-1 develop rapidly. The interference of EHV-1 with innate and adaptive cellular immune responses of the host, and in particular the down-regulation of innate IFN-γ production, takes most likely an active part in decreasing the EHV-1 specific T-cell immunity. An improved definition of protective immunity against EHV-1 paired with advanced, quantitative detection methods for host immune parameter detection seems essential to further mechanistically unravel protection from EHV-1 infection, nasal shedding, as well as viremia. Understanding the interaction of EHV-1 with the host immune system during its various disease outcomes will support the development of effective EHV-1 vaccines and enhance protection from severe EHV-1 outbreaks.

## Supporting information

S1 Table Virus isolation from nasal secretions (PFU/ml) of naïve horses after experimental EHV-1 infection with the EHV-1 strain Ab4 or deletion mutant strain Ab4ΔORF1/71 (n = 5 per group). (DOCX)

S1 Fig Total anti-EHV-1 gB and gD antibodies in nasal secretions of naïve horses (n = 5 per group) after infection with EHV-1 strain Ab4 or its deletion mutant Ab4ΔORF1/71. Non-infected horses were kept as controls. The arrow marks the time of infection. Nasal secretion samples were collected before and at various times after infection. Antibodies were evaluated using an EHV-1 multiplex assay: (A) total anti-gB and (B) total anti-gD antibodies are expressed as median fluorescence intensities (MFI). Mean and standard errors are displayed. Significant differences between groups: a = Ab4 vs. controls, b = Ab4ΔORF1/71 vs. controls, and c = Ab4 vs. Ab4ΔORF1/71.
(TIF)

S2 Fig Anti-EHV-1 gC IgM, IgG3/5 and IgG6 responses in serum from horses (n = 5 per group) after EHV-1 infection with Ab4 or its deletion mutant Ab4ΔORF1/71. Serum antibodies were measured by an EHV-1 multiplex assay and results are expressed as median fluorescence intensities (MFI) for (A) IgM, (B) IgG3/5 and (C) IgG6. The arrow point to the day of infection. Graphs show means and standard errors by group over time. Significant differences between groups are marked: a = Ab4 vs. controls, b = Ab4ΔORF1/71 vs. controls, and c = Ab4 vs. Ab4ΔORF1/71.
(TIF)

S3 Fig Cytokines in serum after infection with EHV-1 Ab4 or Ab4ΔORF1/71. Horses (n = 5 per group) were infected on d0 (arrow). A non-infected control group was included. Serum samples were obtained several times before and after infection. Cytokines and sCD14 were evaluated with fluorescent bead-based assays. Mean and standard errors of (A) sCD14 and (B) IFN-γ in serum are displayed. Significant differences between groups: a = Ab4 vs. controls, b = Ab4ΔORF1/71 vs. controls, and c = Ab4 vs. Ab4ΔORF1/71.
(TIF)
