# Supplementary material for: The deletion of the ORF1 and ORF71 genes reduces virulence of the neuropathogenic EHV-1 strain Ab4 without compromising host immunity in horses
Source: PLoS One. 2018 Nov 15;13(11):e0206679. doi: 10.1371/journal.pone.0206679 (PMC6237298; doi:10.1371/journal.pone.0206679)
Supplement: S1 Table — (DOCX) [file pone.0206679.s001.docx]

**S1 Table. Virus isolation from nasal secretions (PFU/ml) of naïve horses after experimental EHV-1 infection with the EHV-1 strain Ab4 or deletion mutant strain Ab4ΔORF1/71 (n=5 per group).**

| **Group** | **Control** | | | | | **Ab4** | | | | | **Ab4ΔORF1/71** | | | | |
| --- | --- | --- | --- | --- | --- | --- | --- | --- | --- | --- | --- | --- | --- | --- | --- |
| **Horse** | 2 | 6 | 8 | 10 | 14 | 1 | 4 | 5 | 9 | 15 | 3 | 7 | 11 | 12 | 13 |
| d-1 | 0  (no EHV-1 isolated from all control horses at all time points) | | | | | 0 | 0 | 0 | 0 | 0 | 0 | 0 | 0 | 0 | 0 |
| d1pi |  |  |  |  |  | 200 | 300 | 6000 | 300 | 500 | 0 | 0 | 0 | 0 | 200 |
| d2pi |  |  |  |  |  | 0 | 0 | 1500 | 100 | 100 | 0 | 0 | 0 | 0 | 10 |
| d3pi |  |  |  |  |  | 20 | 0 | 100 | 10 | 0 | 0 | 0 | 0 | 0 | 0 |
| d4pi |  |  |  |  |  | 0 | 0 | 10 | 0 | 0 | 0 | 0 | 0 | 0 | 0 |
| d5pi |  |  |  |  |  | 0 | 0 | 0 | 0 | 0 | 0 | 0 | 0 | 0 | 0 |
| d6pi |  |  |  |  |  | 0 | 0 | 0 | 0 | 0 | 0 | 0 | 0 | 0 | 0 |

d-1 = one day prior to EHV-1 infection; pi = post infection
