# Supplementary figures and images for: The deletion of the ORF1 and ORF71 genes reduces virulence of the neuropathogenic EHV-1 strain Ab4 without compromising host immunity in horses
Source: PLoS One. 2018 Nov 15;13(11):e0206679. doi: 10.1371/journal.pone.0206679 (PMC6237298; doi:10.1371/journal.pone.0206679)

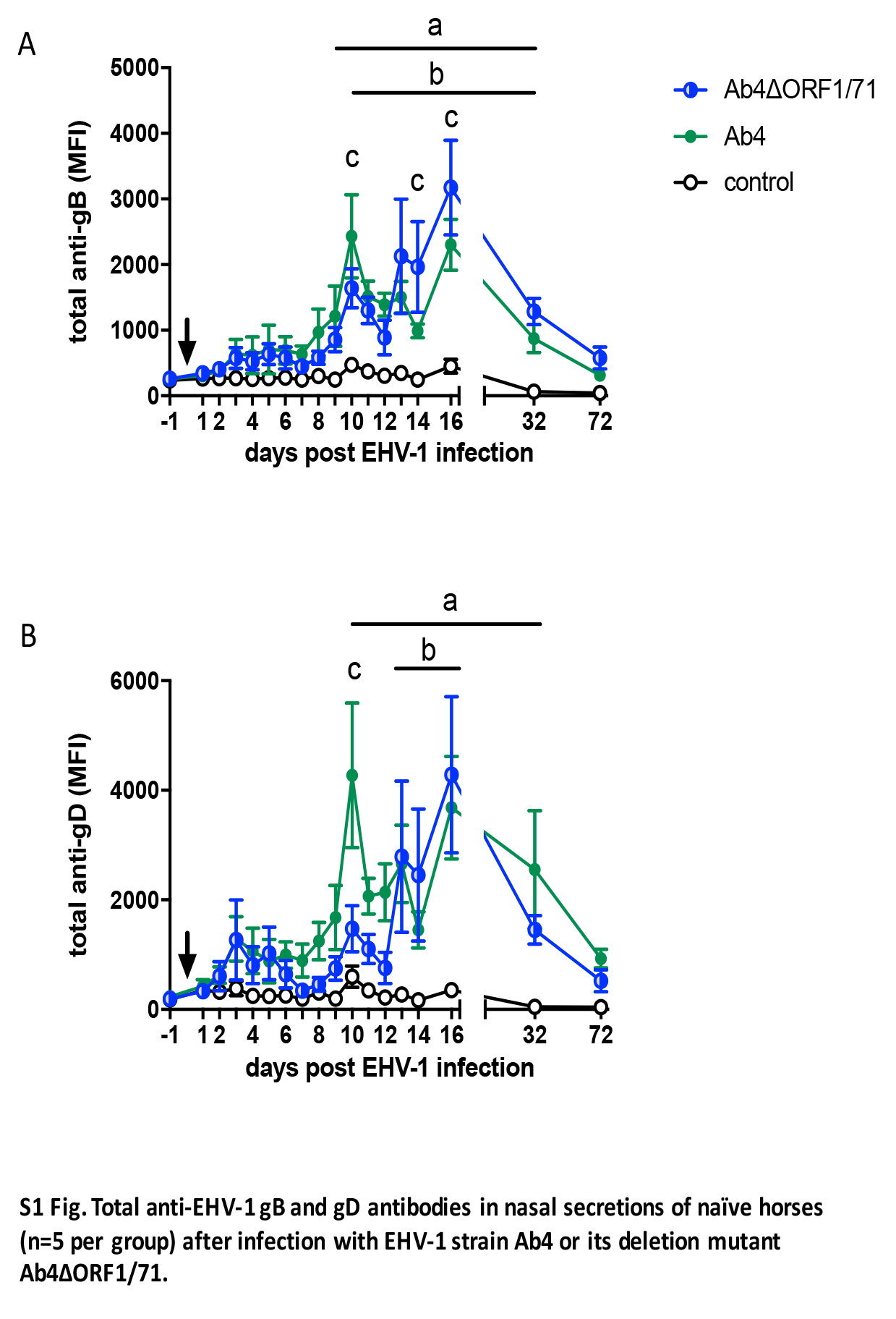

Supplement: S1 Fig — Non-infected horses were kept as controls. The arrow marks the time of infection. Nasal secretion samples were collected before and at various times after infection. Antibodies were evaluated using an EHV-1 multiplex assay: (A) total anti-gB and (B) total anti-gD antibodies are expressed as median fluorescence intensities (MFI). Mean and standard errors are displayed. Significant differences between groups: a = Ab4 vs. controls, b = Ab4ΔORF1/71 vs. controls, and c = Ab4 vs. Ab4ΔORF1/71. (TIF) [file pone.0206679.s002.tif]

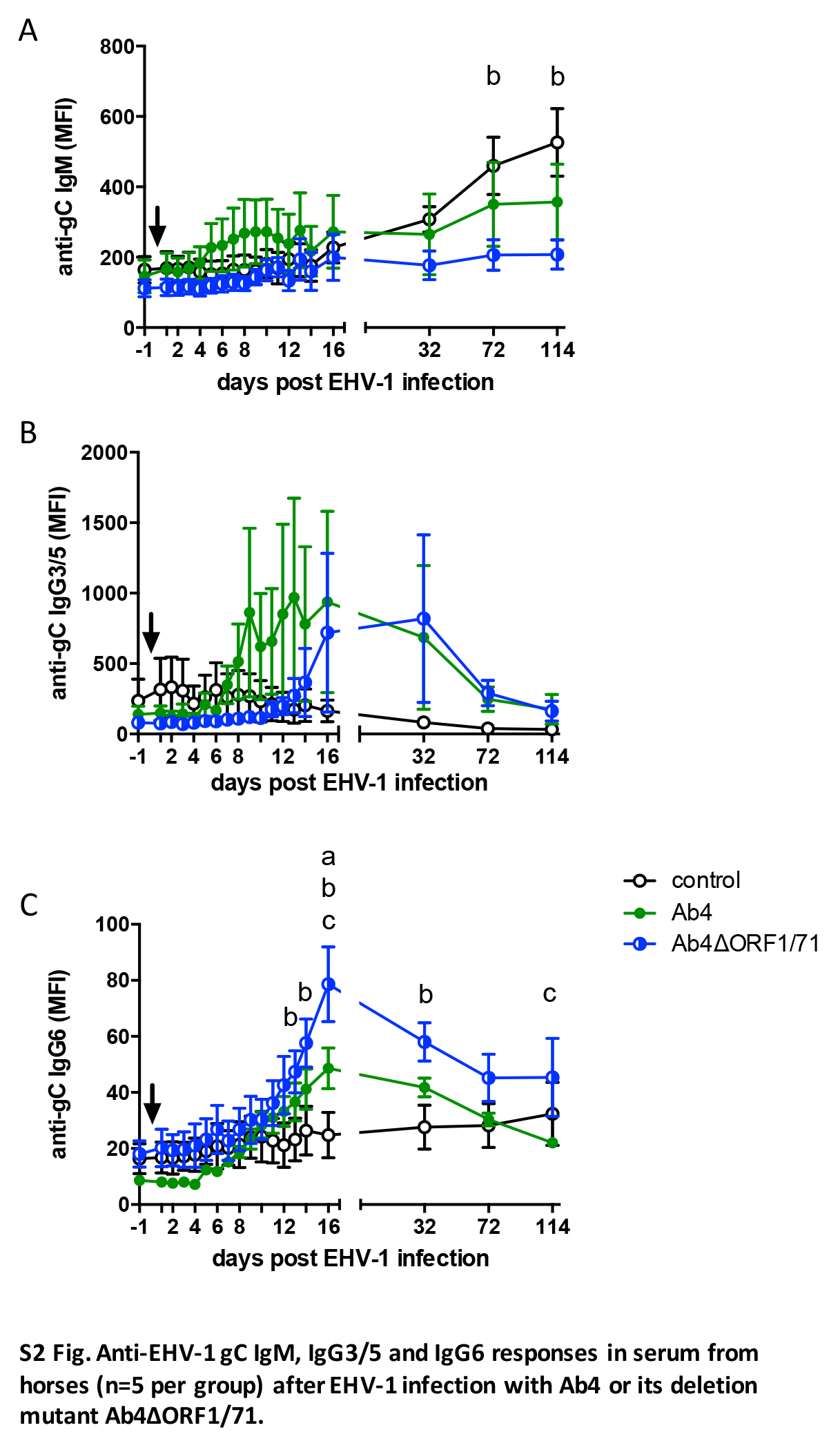

Supplement: S2 Fig — Serum antibodies were measured by an EHV-1 multiplex assay and results are expressed as median fluorescence intensities (MFI) for (A) IgM, (B) IgG3/5 and (C) IgG6. The arrow point to the day of infection. Graphs show means and standard errors by group over time. Significant differences between groups are marked: a = Ab4 vs. controls, b = Ab4ΔORF1/71 vs. controls, and c = Ab4 vs. Ab4ΔORF1/71. (TIF) [file pone.0206679.s003.tif]

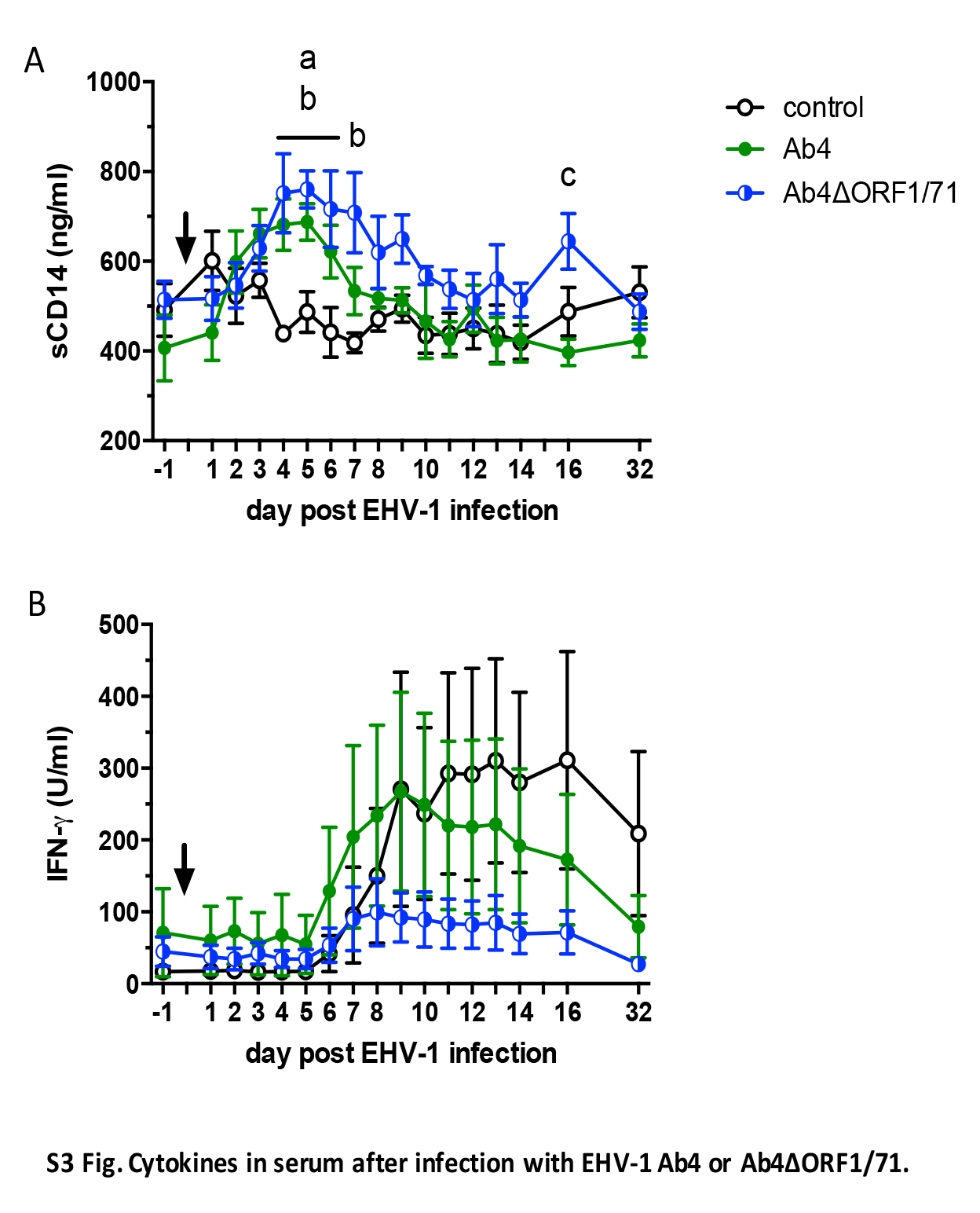

Supplement: S3 Fig — Horses (n = 5 per group) were infected on d0 (arrow). A non-infected control group was included. Serum samples were obtained several times before and after infection. Cytokines and sCD14 were evaluated with fluorescent bead-based assays. Mean and standard errors of (A) sCD14 and (B) IFN-γ in serum are displayed. Significant differences between groups: a = Ab4 vs. controls, b = Ab4ΔORF1/71 vs. controls, and c = Ab4 vs. Ab4ΔORF1/71. (TIF) [file pone.0206679.s004.tif]
